# Gambling-related suicide in East African Community countries: evidence from press media reports

**DOI:** 10.1186/s12889-021-12306-2

**Published:** 2022-01-24

**Authors:** Mark Mohan Kaggwa, Mohammed A. Mamun, Sarah Maria Najjuka, Moses Muwanguzi, Moses Kule, Rahel Nkola, Alain Favina, Raymond Bernard Kihumuro, Gideon Munaru, Innocent Arinaitwe, Godfrey Zari Rukundo, Mark D. Griffiths

**Affiliations:** 1 Department of Psychiatry, Faculty of Medicine, Mbarara University of Science and Technology, Mbarara, Uganda; 2 African Centre for Suicide Prevention and Research, Mbarara, Uganda; 3 CHINTA Research Bangladesh, Savar, Dhaka, 1342 Bangladesh; 4 Department of Public Health and Informatics, Jahangirnagar University, Savar, Dhaka, 1342 Bangladesh; 5 Department of Public Health, Daffodil International University, Dhaka, Bangladesh; 6 Makerere University, College of Health Sciences, Kampala, Uganda; 7 Faculty of Medicine, Mbarara University of Science & Technology, Mbarara, Uganda; 8 Department of psychiatry, Mbarara Regional Referral Hospital, Mbarara, Uganda; 9 Department of Psychiatry, Bugando Medical Center, Mwanza, Tanzania; 10 Kisii Teaching and Referral Hospital, Kisii, Kenya; 11 Psychology Department, Nottingham Trent University, 50 Shakespeare Street, Nottingham, NG1 4FQ UK

**Keywords:** Gambling, Betting, Uganda, Kenya, East Africa, Suicide, Media reporting suicide, Gambling-related suicide, East African community, Gambling laws

## Abstract

**Background:**

Gambling activities and associated mental health problems have become a topic of increased concern globally. Many individuals with a severe gambling disorder have gambling-related suicidality. However, no study has explored gambling-related suicide in East African Community (EAC) countries. The present study investigated the press media reporting of gambling-related suicide cases from EAC countries.

**Methods:**

As there is no established suicide database in that region, media reports were utilized to collect gambling-related suicide data. Gambling-related suicide case reports were searched for in EAC countries’ press media websites using *Google*. After removing duplicates, a total of 18 suicides were found.

**Results:**

The victims were all males aged 16 to 40 years. The most prevalent reason for the death was university students who had used their university tuition fees for gambling and losing the money (*n* = 4/17). All the suicide deaths were in Kenya (10/18), Uganda (7/18), and Tanzania (1/18). Betting on soccer was the most common type of gambling reported (*n* = 11/15), and hanging was the most used mode of suicide (*n* = 10/16).

**Conclusions:**

Based on the press media reports, 18 males were identified as having carried out gambling-related suicides. The countries with the most widespread opportunities to gamble had more gambling-related suicides, although the number of suicides was very small.

## Introduction

Gambling is risking of money (or something of financial value) on an event at least partly determined by chance in the hope of winning something of higher financial value [1]. Worldwide, 26% of the population actively gamble [2]. Sports events have created a fertile ground for sports betting due to the popularity of games and the uncertainty of outcomes [3]. For decades, gambling has been traditionally been engaged in at designated venues such as bookmakers, casinos, among others, but has now moved to digital platforms [4]. The introduction of online gambling, esports, and betting via mobile devices such as smartphones is of potential concern to susceptible populations [3]. In sub-Saharan Africa, gambling is perceived as a source of income for many young people, and gambling, for this reason, may be a contributory factor for gambling disorder (GD) [3].

GD is a condition that describes a gambler’s inability to control their gambling despite the detrimental consequences of the behavior [5, 6]. Globally, the prevalence of GD ranges between 0.12 to 5.8% [7]. In addition, evidence from several studies conducted in Africa has shown the increasing numbers of youths reporting gambling-related problems, including financial difficulties (e.g., chronic poverty), domestic violence, family breakups, criminality, and mental health disorders (e.g., depression, anxiety, stress) [4, 8, 9]. However, to date, no African study has explored gambling-related suicide.

In East African Community (EAC) countries (comprising Uganda, Kenya, Tanzania, Burundi, Rwanda, and South Sudan), GD has become a growing public health concern. A study conducted in Uganda found 24.3% of individuals in an urban center gambled, with 20% involved in sports betting, with the main motivation for gambling in the whole sample being to win money (76.6%) [10]. Effects such as family dysfunction, divorce, alcohol, and substance use have been associated with gambling [11]. Also, similar issues have started to occur among neighbors to EAC countries, such as Malawi, where the odds ratios of alcohol and substance abuse, involvement in physical fights, theft, and vandalism were 4.17, 2.26, 3.26, and 3.96 times higher among gamblers, respectively [12]. In Uganda, evidence suggests that problem gambling is associated with mental health problems such as depression, anxiety, and suicidality [13]. Another study in Uganda reported 21 cases of suicide among university students, five of which were gambling-related [14].

Studies on gambling-related suicide in EAC countries are few, and there is no suicide database in these countries [15]. Therefore, the present study adopts a similar approach used by many researchers in other countries without suicide databases by utilizing media reports to investigate gambling-related suicide in EAC countries [14, 16–19].

## Literature review

### Gambling in EAC countries

The EAC is a regional intergovernmental organization of six partner states whose activities are guided by its treaty that established the community [20]. This is one of Africa’s fastest economically growing regions, involved in many high tax-generating activities such as gambling [20]. EAC countries’ gambling activities are highest in the continent, with 76% in Kenya and 57% of those aged 17–35 years in Uganda having previously engaged in gambling at some point in their lives [9, 21]. EAC countries were among the first in Africa to legalize all forms of gambling immediately after their independence (late 1960s) [22–24]. These countries have legalized various types of gambling, such as casinos, sports betting, pool games, bingo, scratch-cards, slot machines, table games, and lotteries [8, 11] meaning that most forms of gambling are legally available in EAC countries.

Gambling activities generate large amounts of income for the governments in the region and contribute to the gross domestic product (GDP) through income taxes and other taxes such as taxes on using ‘mobile money’ (a technology that allows individuals to receive, store, and spend money using a mobile phone) through the telecom companies and other taxes such as ‘pay-as-you-earn’ through the employees of the companies [8, 10, 25]. For example, the tax revenues generated from the gambling industry in Uganda increased from UGX 0.24 billion (approximately $US 67,729) in 2002/3 to UGX 11.1 billion (approximately $US 3,132,447) in 2013/14 [10]. Due to their benefits to the government and the gambling companies, gambling services are currently available to all regions in EAC countries as long as there is an accessible mobile phone network. In addition, digitalization through mobile money and advanced marketing strategies to convince youth to gamble to ‘get rich’ has turned gambling into a perceived potential source of income in the region [26]. However, all these countries have set laws and regulations to limit the hazards posed by gambling activities.

### Gambling laws and regulations in EAC countries

With the exception of Burundi and South Sudan, all EAC countries have legalized gambling and have rules and laws governing gambling. However, none of the countries has regulations concerning the legalization of online gambling. The most updated laws and regulations on gambling are in Kenya (i.e., Gambling Act 2020), and Rwanda has the oldest (Law No. 58 of 2011). The legal age of gambling is 18 years and above, apart from Uganda, where it was set at 25 years and above in 2016, and failure to comply results in the companies paying a fine of $US 300 every time someone underage is caught gambling. All EAC countries prohibit the selling or the use of addictive substances such as cigarettes and alcohol within the gambling premises. Tanzania banned advertisements by gambling companies. The other EAC countries have no specific taxes or methods to control advertisements from gambling companies. The EAC countries try to maximize taxes, with each winner paying 20% of the amount won to the government. For details, see Table 1.

**Table 1:** Rules and regulations of gambling in the different East African countries

	Law/act	Board in governing gambling	Legal age for gambling	Substances of abuse	Advertisement	Tax on winnings	Opening and Licensing cost	Online gambling
Kenya	Gambling act 2020	Betting Control and Licensing Board	18 years for gambling and 16 years for lotteries	Prohibited in gambling premises	An extra 35% charged on gambling advertisements	20%	$44,000 for local companies and $50,950 for foreign companies	Legal (no specific regulations)
Uganda	The lotteries and gaming act of 2016	National Lotteries and Gaming Regulatory	25 years	Prohibited	Act prohibits advertising of unauthorized gaming and betting pools	20%	License to establish or operate is $70,600 except for casinos and lotteries	Legal (no specific regulations)
Tanzania	Sports betting rules 2016	Gaming Board of Tanzania	18 years	Prohibited	Ban on advertisements	20% (except for casino winnings, 12%)	$300,000 for local companies and $5000,00 for foreign companies	Legal (no specific regulations)
Rwanda	Rwandan gambling legislation 2011	Rwanda Gambling Board	18 years	No laws	No laws	No tax laws	$1504	No laws

### Complications of gambling

There is a well-established association between problem gambling and divorce, childhood maltreatment, crime, addiction to various psychoactive substances, and family dysfunction [11, 27–33]. Gamblers’ family members report high conflict and violence, sexual displeasure, anger, loss of personal money and/or household property, lies and deception, and separation/divorce [28, 30, 32, 34]. Social harms and antisocial behaviors are also frequently reported by problem gamblers [28]. Gamblers may borrow money either legally or illegally, leading them and/or their families into heavy loans or financial losses, resulting in failure to meet family financial needs.

The effects of taking out loans and loss of money may lead to severe alcohol dependence, drug abuse, suicidal ideation, irritability, depression, anxiety, stress, and sleep deprivation [11]. However, despite the associations between problem gambling and many detrimental behaviors and negative psychological impacts, only seven studies in EAC have explored the consequences of problem gambling [4, 8–11, 26, 35]. Six have been in Uganda and one in Rwanda, leaving out other highly affected countries in the region like Kenya. In addition, a recent publication by Kaggwa et al. (2021) reported 5 out of 21 university students allegedly died by suicide after using money meant for university tuition fees on gambling [14]. Therefore, gambling-related suicide may be a concern among youths in the East Africa region.

### Suicide and gambling

Several studies have reported an association between gambling and suicidality [36–39]. However, several studies on gambling and suicidal behavior have focused on non-fatal suicide attempts or suicidal ideation, and have consistently reported high rates of suicidal ideation or suicide attempts among individuals with problem gambling compared with the general population [40–45]. For example, one study reported that the prevalence of suicidality among gamblers was as high as 49.2% for suicidal ideation and 18% for suicide attempts, compared to 25.8 and 7.9% among participants without GD [44]. The triggers for suicidal ideation and attempts among individuals with GD include financial debts, family and social difficulties, legal and employment problems, and the psychological distress (e.g., depressive symptoms) that usually co-occur with the disorder [45–47]. Factors associated with problem gambling-related suicidality include depression, cluster B personality disorders, alcohol dependence, and male gender [42, 46–50]. Despite the consistent association between gambling and suicidality, with arguably high numbers of individuals reporting suicidal ideation, plans, and attempts, fewer than 10% are estimated to seek help for their problem gambling-related suicide [51, 52].

Confirming that suicide is problem gambling-related is challenging since there are likely to have been multiple factors involved. Getting corroborative evidence from family, close friends, and neighbors is therefore important in determining if suicide was due to problem gambling. This process is known as a psychological autopsy [53]. The method makes data more accurate in confirming causality. However, to date, only one study has employed such a method [38]. They found that of the 1201 suicide cases in 2003 in Hong Kong, 19.4% were due to gambling, and the failure to pay debts at the time of death was the reason given for almost half the gambling-related suicides [38]. Media reports also sometimes provide a proxy for a psychological autopsy because they report collateral information from family, friends and/or witnesses. In the present study, the press media reports were collated to investigate gambling-related suicide in EAC countries.

## Methods

### Search strategy

Content analysis of press media reports focusing on gambling-related suicide was used because no East African countries have a suicide database. Such media reports often include information provided by the family, friends, neighbors, and/or others who knew the deceased and therefore has some information that resembles a psychological autopsy [53]. It is also a method employed in other studies in countries where there are no suicide databases such as Bangladesh, India, Uganda, and Pakistan [14, 16–19]. Utilizing this method, data were searched from different press/media websites in each of the six EAC countries (Uganda, Kenya, Tanzania, Burundi, South Sudan, Rwanda) in late June 2021. The time period for collecting data was from January 2014 (the first reported gambling suicide in EAC countries) to June 2021. The following keywords were used in the search: ‘suicide’ (OR its translation depending on the language of the media house) AND ‘betting’ or ‘gambling’. An additional search was done on *Google* to identify additional cases, and the following keywords were used: ‘suicide’ AND ‘betting’ OR ‘gambling’ AND each EAC country.

### Media article selection criteria

All articles about suicide in the different media houses were read, and those that directly reported betting/gambling to be the cause of suicide were included in the present study.

### Data extraction

Information extracted from the media article included: (i) URL of the article, (ii) when the suicide occurred, (iii) country in which the suicide occurred, (iv) gender of victim (v) age of victim, (vi) level of education of victim, (vii) marital status of victim, (viii) amount of money lost gambling prior to suicide, (ix) employment status of victim, (x) mode of suicide, (xi) location of suicide, (xii) suicide note presence or absence, (xiii) type of gambling involved in prior to suicide, and (xiv) reason for suicide. The final data extracted are presented in Table 2.

**Table 2:** Gambling Suicide Victim’s Characteristics

Case	Year	Country	Day and time	Age	Education level	Marital status	Money lost (USD)	Employment	Mode of suicide	Location of suicide	Suicide note	Type of betting	Reported reason for suicide
1 [54]	May, 2014	Uganda	Friday, day	–	Tertiary	–	–	University student	Poison ingestion	Banana Plantation (Garden)	Yes	Soccer betting	Betting with his university tuition fees
2 [55]	December, 2014	Uganda	Friday, day	30	Tertiary	–	195	–	Poison ingestion	Garden	No	Card games	Disappointed with the loss
3 [56, 57]	February, 2015	Uganda	Friday, day	–	–	–	139	Boda boda rider	Hanging	Home	Yes	Soccer betting (Arsenal loss to Monaco – European champion League)	Used his employer’s money to bet, then lost (reportedly depressed)
4 [58]	2016	Kenya	–	–	Tertiary	Never married	790	–	Hanging	Hostel	No	Soccer betting	Losing money on a bet
5 [59]	April, 2016	Kenya	–	–	–	–	436	–	Hanging	–	–	Soccer betting	–
6 [59–61]	July, 2016	Kenya	Wednesday	28	Tertiary	Never married	741	University student	Hanging	Home	Yes	Soccer betting (Germany vs. Italy Euro game 2016)	Bet his college tuition fees and lost.
7 [62]	August, 2016	Kenya	Wednesday	–	–	–	417	–	–	Home	No	Soccer betting (*SportPesa*) – Online	Borrowed the money the Bank, placed all the stake on Real Madrid, and lost
8 [63, 64]	September, 2016	Uganda	Thursday	–	Tertiary	–	34	University student	Poison ingestion. One report (Tusiime, 2016) says attempted suicide and a later report says actual suicide (Ssebwami (2019)	Hostel	No	Soccer betting – Landbased betting store	Used his tuition fees for betting
9 [65, 66]	March, 2017	Kenya	Thursdaynight	30	–	Married with 2 children	84	Businessman who used to sell compact discs	Hanging	Home	No	Soccer betting	Failure to win. Depended on the money to pay off debts i.e., bank loan and rent
10 [67, 68]	May, 2018	Tanzania	Saturday, night	–	Tertiary	–	–	University student	Hanging	Hostel	No	Soccer betting	Losing a bet in Liverpool vs. Madrid match (2018 Champions League final)
11 [69]	September, 2018	Kenya	Saturday	–	–	–	556	–	–	Home	No	Soccer betting – Online	Lost money while in-play betting on a Ghana vs. Kenya match
12 [70]	September, 2018	Uganda (Indian natives)	Saturday, night	23	Tertiary	–	13,889	A salesman of a distiller’s company	Hanging	Hotel	No	–	Lost company’s money in betting and had accumulated hotel bills
13 [71]	November, 2017	Uganda	Wednesday	–	Tertiary	Married	28	Secondary school teacher	Hanging	Behind mother home	Yes	Soccer betting	Misused student tuition for betting (i.e., lost money during Uganda win vs. Congo-Brazzaville in a World Cup qualification game)
14 [72, 73]	December, 2018	Kenya	Wednesday, day	25	Tertiary	Married	–	Factory worker	Drowning	Home	No	–	Long standing losses due to betting (betting mission goes sour)
15 [74]	2018	Uganda	Saturday	16	Secondary	Never married	–	Student	Hanging	Hostel – in his bed room	No	Casino – land-based	Possibly misused tuition fees for gambling in a casino
16 [75]	2018	Kenya	–	32	–	Never married	–	Caretaker of a block of houses	Hanging	Home	No	Soccer betting	Allegedly hunted down by creditors, hanged self after losing a bet-which he had hoped could help him clear his debts.
17 [76, 77]	December, 2019	Kenya	Wednesday, night	27	Tertiary	–	–	Worked at Kenya Revenue Authority	Carbon monoxide poisoning	Rental	Yes	–	Unsuccessful bets
18 [77–80]	January, 2020	Kenya	Monday, day	40	–	Married	138,889	–	Jumping from a height (police allegedly also found some tablets in the deceased’s pocket)	At the casino	No	Casino – land-based	Losing money while gambling at a casino

### Quality control

The research team included individuals from different EAC countries, fluent in the languages used by the different press/media in the countries (i.e., Lou, Luganda, Kiswahili, French, Kinyarwanda, Kirundi, Arabic, and Dinkah). Records with the same URL, date of death, name, age, mode of suicide, and money lost during the bet were considered duplicates, and only one entry was considered. Information from different websites (i.e., with different URLs) was used to supplement the information or confirm the information reported.

## Results

A total of 18 gambling-related suicides were collected, with the majority being from Kenya (10/18), followed by Uganda (7/18) and Tanzania (1/18), while the rest of the East African countries had no reported suicide-related to gambling. The suicides occurred from 2014 to 2020, with the majority occurring in 2018 (6/18) and 2016 (5/18). Most deaths occurred in the last quarter of the year. All participants were below the age of 40 years, and the youngest was aged 16 years. Two Ugandan gambling-related suicide victims were below the legalized age for gambling in the country (< 25-years). The majority of the participants had tertiary/university levels of education (10/11) and were never married (4/8). Seven victims were reported to be employed (Table 2). (Please note that where the denominator is less than 18 in the reported cases, this means that such information was not explicitly mentioned in the media report).

The deceased were most commonly involved in betting on soccer matches (*n* = 11/14), and two were casino gamblers (2/16). Only two individuals gambled online. The total amount lost in gambling by the deceased was the equivalent of $US 156,197, with the mean amount lost gambling immediately before death was $US 342, a median of $US 396, and ranged from $US 28 to $US 138,889. Hanging was the most common method of suicide (10/17), followed by poisoning (3/17). Suicide most commonly occurred at individuals’ homes (15/18), and the most common reason for suicide following gambling was the use of money for university tuition fees for gambling and losing it (4/17). Only one individual was reported to have been experiencing depression prior to suicide, and five individuals left a suicide note (Table 2).

## Discussion

The present study investigated the victims of gambling-related suicide in the six East African Community (EAC) countries using data from the press media reports. The EAC is a region with multiple gambling companies that heavily invest in persuasive gambling advertisements claimed by some to get more youth to gamble [81]. Gambling advertisements have been reported to increase gambling behaviors among sports fans [82]. However, the present study was not able to infer causality due to the descriptive nature of the results. The following subsections discuss the profiles of the suicide victims.

### Country

Kenya had the highest number of gambling-related suicides, which might possibly be because Kenya has one of the most popular and used betting companies in the continent (*SportPesa*) [81], and has the highest number of gambling companies (40 casinos and 89 registered betting companies) followed by Uganda (16 casinos and 67 registered betting companies) [23, 83]. Therefore, this increased opportunity arguably puts more individuals at a higher risk of problem gambling compared to other EAC countries, and with increased problem gambling, there may be an increased incidence of problem gambling-related suicides. These figures should also be considered in relation to the suicide rates in each of these countries. More specifically, the suicide rates for different EAC countries per 100,000 individuals are 6.2 for Burundi, 6.1 for Kenya, 5.6 for Rwanda, 4.6 for Uganda, 4.3 for Tanzania, and 3.9 for South Sudan [84].

### Demographics of the victims

All the victims identified in the present study were male, which is unsurprising given that historically, males have been reported to die more by suicide than females and are more heavily involved in gambling than females [35, 42, 85]. In addition, males show more positive attitudes towards gambling than females [86–89]. This may be because gambling is considered a more masculine activity [90]. However, recent studies have shown an increased involvement by females in online lotteries and online gambling [10, 88, 91–93].

The present study found that all the suicide victims were below the age of 40 years, reflecting the different countries’ demographic characteristics; for example, a large proportion of the populations in EAC countries are below the age of 25 years based on the most recent census data: 68% in Uganda, 64% in Tanzania, 62% in Kenya, and 60% in Rwanda [94]. Moreover, youths are at a greater risk of both GD and suicidality [95]. In addition, some of the gambling-related suicides may be compounded by the high level of poverty in the region. Almost all the EAC countries are in the low-income countries’ economy categories, apart from Tanzania, a middle-income country [96]. This is expected to get worse due to the COVID-19 pandemic in the region [97]. The majority of the individuals in East Africa survive on less than $US 2 a day and remain consistently supported by subsistence forms of farming or boda-boda riding (bicycle or motorcycle taxis common in EAC countries), a less lucrative form of business making individuals look for easier ways to earn a living like gambling [10]. A study carried out among Ugandan boda-boda men showed that low-income earners who have lots of free time with inadequate supervision from their employers are more likely to engage in gambling [35]. This increases the risk of GD and may increase the risk for gambling-related suicide.

Due to poverty and low earnings in EAC countries, many families invest heavily in their children to have any form of education to increase the chances of family support through children’s economic success [98]. However, this comes with multiple challenges, such as the inability to have basic needs at school, such as pocket money, and many students end up gambling to earn extra money [10]. Moreover, even after successfully finishing school, it is difficult to get employed. It has been claimed that gambling has become a dominant potential source of income for most unemployed youths [10]. Therefore, it is unsurprising that the majority of the gambling-related suicides were among unemployed individuals in the present study. Previous studies have found a greater number of gambling-related suicides among unemployed individuals than those employed [26]. However, even for those who get a job, the minimum wages per year are very low (i.e., $US 830 for Kenya, $US 95 for Uganda, and $US 1593 for Tanzania) to sustain themselves and their families, making some individuals resort to gambling to increase their income [99].

### Gambling activities

The majority of the suicide victims in the present study were involved in betting on soccer matches, a popular type of betting in East Africa. It may be attributed to the fact that most EAC countries were former British colonies, and many individuals are attached to watching the English Premier League. In addition, many Premier League teams are sponsored by betting companies [35, 100]. This makes betting an acceptable activity, and many poor youths, especially students, are attracted to betting [10]. In addition, there are many additional attractive options, such as a chance to win the opportunity to go and watch actual Premier League football games in England.

### Suicide-related characteristics

Hanging is a common method used by individuals who die by suicide, especially among victims reported in press media report studies [14, 16–19]. This may be a possible bias in press media reporting due to the traumatic nature of individuals who hang themselves, which attracts media attention. However, other modes of suicide were reported in the present study, such as jumping from a great height and drowning (Table 2). Suicides occurred more at particular periods, such as major sporting tournaments in the world, including the soccer World Cup in 2018 (6/18) and soccer Euros 2016 (5/18). This could be due to the extensive advertisements during these periods, and the majority of the individuals in EAC enjoy watching soccer [35]. These advertisements continuously make gambling an acceptable activity, and a minority of vulnerable individuals may end up dying by suicide after losing heavily.

## Study strengths and limitations

One of the key strengths of the study was the diverse language groups of the authors enabling the reading of a wide selection of of media reports. Moreover, many of the authors reside in different countries of the EAC and are therefore aware of the cross-cultural differences in the region which were important in data interpretation. Despite these strengths, numerous limitations should be considered when interpreting the findings. First, the form of gambling reported in each case may not have been the only form of gambling with which the individual was engaged (i.e., many gamblers engage in more than one type of gambling). Secondly, not every gambling-related suicide would necessarily have been reported by the media and therefore this is likely to underestimate the true incidence of gambling-related suicides. Third, there may be a bias in which types of gambling-related suicides are reported by the media (e.g., a tendency to report the suicides of young, well-educated men as opposed to other demographic groups as these may be deemed by editors to be more ‘newsworthy’). Consequently, media reports may only be reporting a minority of actual gambling-related suicides. Fourth, the data collected are totally reliant on what was reported and the information may not necessarily be totally accurate (e.g., the total amount of money lost gambling by the individual, the gambling pattern of the individual, the exact cause of death, etc.). Fifth, some of the information in the press reports may have been omitted due to reporters following guidelines of how suicides should be reported in the media (e.g., World Health Organization) given the research showing an increase in suicides and suicide attempts following media reporting. Sixth, the search strategy only included ‘gambling’ and ‘betting’ as these are the most commonly used words in the region to describe the focus of the present study. Other words such as ‘wagering’ were not included in the search and therefore there is a small possibility that some cases may have been missed.

## Conclusions

Suicide is a complex multifactorial issue, where its completion consists of a combination of more than one factor, including biological, psychological, and environmental factors. Assessing the alleged suicide stressors from the press media reports can be useful because the EAC countries lack national suicide databases to retrieve information about suicide cases. Based on the press media reports, 18 males in the present study were identified as victims of gambling-related suicides. Moreover, the countries with the most widespread opportunities to gamble (i.e., Kenya and Uganda) had more gambling-related suicides, although the number of suicides as a percentage of the total population was very small and any causal association should be treated with caution given other factors are important (e.g., gambling participation rates in each country).

## Data Availability

The datasets will be made available to appropriate academic parties on request from the MMK.

## Abbreviations

BCLB: Betting Control and Licensing Board
COVID-19: Coronavirus disease
EAC: East African Community
GD: Gambling Disorder
GDP: Gross Domestic Product
UGX: Uganda Shillings
URL: Uniform Resource Locator
US: United states dollars
