# Correction to: Gambling-related suicide in East African Community countries: evidence from press media reports

**DOI:** 10.1186/s12889-022-12691-2

**Published:** 2022-02-08

**Authors:** Mark Mohan Kaggwa, Mohammed A. Mamun, Sarah Maria Najjuka, Moses Muwanguzi, Moses Kule, Rahel Nkola, Alain Favina, Raymond Bernard Kihumuro, Gideon Munaru, Innocent Arinaitwe, Godfrey Zari Rukundo, Mark D. Griffiths

**Affiliations:** 1 Department of Psychiatry, Faculty of Medicine, Mbarara University of Science and Technology, Mbarara, Uganda; 2 African Centre for Suicide Prevention and Research, Mbarara, Uganda; 3 CHINTA Research Bangladesh, Savar, Dhaka 1342 Bangladesh; 4 Department of Public Health and Informatics, Jahangirnagar University, Savar, Dhaka 1342 Bangladesh; 5 Department of Public Health, Daffodil International University, Dhaka, Bangladesh; 6 College of Health Sciences, Makerere University, Kampala, Uganda; 7 Faculty of Medicine, Mbarara University of Science & Technology, Mbarara, Uganda; 8 Department of psychiatry, Mbarara Regional Referral Hospital, Mbarara, Uganda; 9 Department of Psychiatry, Bugando Medical Center, Mwanza, Tanzania; 10 Kisii Teaching and Referral Hospital, Kisii, Kenya; 11 Psychology Department, Nottingham Trent University, 50 Shakespeare Street, Nottingham, NG1 4FQ UK

**Correction to: BMC Public Health 22, 158 (2022)**

**https://doi.org/10.1186/s12889-021-12306-2**

Following publication of the original article [1], the authors would like to add an affiliation and correct the error in the author name of Mohammed A. Mamun.

The incorrect author name is: Mohammed A. Mamum

The correct author name is: Mohammed A. Mamun

The added affiliation is: Department of Public Health, Daffodil International University, Dhaka, Bangladesh.

The author group has been updated above and the original article [1] has been corrected.
